# Changes in blood flow distribution after hypogastric artery embolization and the ischaemic tolerance of the pelvic circulation

**DOI:** 10.1097/MD.0000000000014214

**Published:** 2019-02-01

**Authors:** Jun Nitta, Katsuyuki Hoshina, Toshihiko Isaji

**Affiliations:** Division of Vascular Surgery, Department of Surgery, The University of Tokyo, Tokyo, Japan.

**Keywords:** 3D imaging, buttock claudication, collateral vessels, computed tomography, hypogastric artery

## Abstract

This study aimed to compare the pelvic cavity vasculature before and after the interventional occlusion of a hypogastric artery (IOHA) and to reveal the protective mechanism of the collateral vessels against pelvic ischaemia.

Sixty-nine patients with abdominal aortic or aortoiliac aneurysms who underwent endovascular aneurysm repair accompanied with IOHA were retrospectively analysed. Patients were divided into those who complained of buttock claudication (BC) group and asymptomatic patients (non-BC group).

Two analyses were performed. In Study 1, the factors associated with postoperative BC were evaluated in patients who underwent IOHA using only 0.035 Tornade embolization coils. In Study 2, the pelvic arterial volume (PAV) was assessed in patients with both pre- and postoperative multidetector computed tomography images. PAV was calculated by subtracting the aortoiliac artery volume from the total PAV. The PAV ratio was defined as the postoperative PAV divided by preoperative PAV and represented collateral development in the pelvis.

In Study 1, BC occurred in 16 patients (BC group) and did not occur in 25 patients (non-BC group). Significantly more coils were used in the BC group than in the non-BC group (8.6 ± 1.0 vs 5.6 ± 0.83, *P* = .013). Study 2 had 24 patients in the BC group and 31 patients in the non-BC group. The PAV ratio was significantly higher in the BC group than in the non-BC group (0.93 ± 0.05 vs 0.62 ± 0.04, *P*<.0001).

The use of more coils in IOHA is associated with BC. In addition, volumetric analysis revealed that less collateral vessel development occurred in the non-BC group than in the BC group, which might reflect a potential reservation capacity of non-BC patients for acute pelvic ischaemia.

## 1 Introduction

Interventional occlusion of a hypogastric artery (IOHA) is sometimes performed in patients who undergo endovascular aneurysm repair (EVAR) for abdominal aortic aneurysms accompanied with an iliac aneurysm or a short common iliac artery. The most frequent adverse effect of acute pelvic ischaemia due to IOHA is buttock claudication (BC), and its rare but severe complications include skin necrosis, colon ischaemia, and paraplegia.^[1,2]^

Although BC is not fatal and is usually resolved in the chronic state, its incidence is high and the post-EVAR quality of life is impaired.^[2]^ Interestingly, the incidence varies greatly in the literature, from 22% to 55%.^[1–7]^ This variation may be due to difficulties in the evaluation of BC, such as poor clinical assessment criteria, lack of prospective control data, and possible confusion with common mobility-limiting conditions in the target population.^[2]^ Complaints of BC are sometimes overlooked because of its benign and non-fatal nature; therefore, the reported incidence of BC may be greater when detailed information regarding BC is specifically gathered from patients who underwent IOHA, such as by email or telephone interviews.^[6]^ If evaluated accurately, BC may be considered a good indicator of tolerance to pelvic ischaemia.

Pelvic circulation is mainly maintained via the flow of the mesenteric, hypogastric, and femoral arteries. We sometimes accidentally found occlusion of these arteries in elderly patients with atherosclerotic predisposition. However, very few patients exhibit ischaemic symptoms. Although IOHA causes acute pelvic ischaemia, which may result in BC, in most cases, the symptoms are resolved afterwards. We assume that this resolution of symptoms is due to the development of collateral vessels in the pelvic cavity.

Although a few reported methods exist for analysing the haemodynamic alterations after IOHA, such as the penile-pressure measurement,^[8,9]^ these assessments do not correspond directly to the alterations. In contrast, an analysis of the changes in the vascular volume of the pelvic cavity would be more direct and informative. Therefore, the purpose of this study was to compare the vasculature in the pelvic cavity before and after the IOHA and to reveal the protective mechanism of collateral vessels against pelvic ischaemia. We hypothesised that collateral vessels would develop after the interventional occlusion to compensate for the decreased pelvic flow and would be more pronounced in asymptomatic patients.

## 2 Methods

### 2.1 Patients and interventional strategy

This retrospective study was approved by our institutional research ethics committee (approval no. 3316 (3)), and all patients provided written informed consent for participation. A total of 69 patients with abdominal aortic or aortoiliac aneurysms who underwent EVAR accompanied with IOHA because of anatomical conditions (iliac dilatation or a short common iliac artery inappropriate for stent graft landing) in our department from January 2012 to November 2018 were included.

The IOHA was performed before EVAR using the following strategy. A maximum of five 0.035 Tornade embolization coils (Cook Medical Inc., Bloomington, IN, USA; 6–10 mm) were used for the main stem of the hypogastric artery (HA) while preserving the communication of the distal branches. Occasionally, additional coils were used for distal branches, until a delayed flow into the HA was observed. The interventional occlusion into the distal branches of the HA was defined as distal IOHA and that of the main stem of the HA was defined as proximal IOHA. For cases requiring bilateral IOHA, the interval between the initial IOHA and EVAR and the contralateral IOHA was at least 2 weeks.

Postoperative symptoms were determined in an interview performed by each operator. BC was diagnosed when the patients complained of buttock pain within 5 min or less than 200 m of continuous walking. The patients were divided into 2 groups: patients who complained of BC (BC group) and asymptomatic patients (non-BC group). In this study, 2 analyses were performed.

### 2.2 Study 1

In Study 1, we comparatively analysed patients who underwent IOHA using only the 0.035 Tornade embolization coils. Thus, 28 patients were excluded: AZUR hydrocoils (Terumo Corp., Tokyo, Japan) were used in 3 patients, 0.018 Tornado embolization coils were used in 1 patient, 0.018 Interlock was used in 1 patient, MReye embolization coils (Cook Medical Inc.) were used in 5 patients, and AMPLATZER vascular coils (St. Jude Medical, LLC, MN) were used in 17 patients, and in 1 patient, the HA orifice was sealed without coils.

### 2.3 Study 2

In Study 2, a volumetric analysis of the vessels in the pelvic cavity was performed. Patients who underwent both preoperative and postoperative multidetector computed tomography (MDCT) with slices of less than 5 mm were included. Thus, 14 patients were excluded: 10 patients did not undergo enhanced MDCT because of renal failure, 1 patient developed an acute stent graft occlusion on postoperative day 4 and 3 patients had poor-resolution MDCT images.

### 2.4 Image analysis

**Figure 1:**
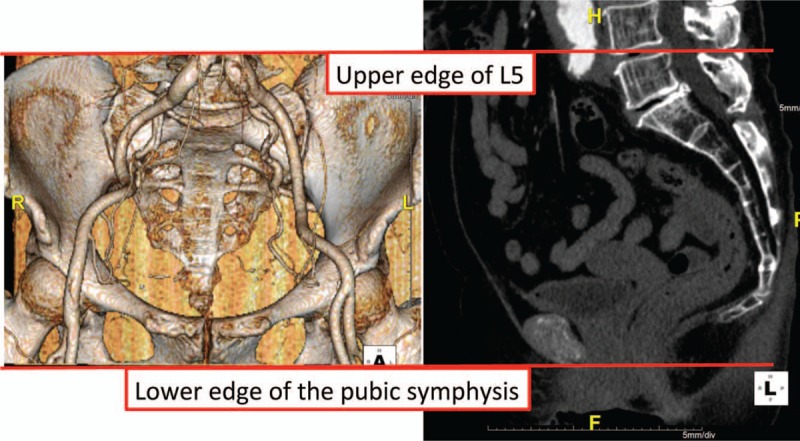
The range of the pelvic cavity region of interest (from the upper edge of L5 to the lower edge of the pubic symphysis).

Patients in Study 2 underwent MDCT before the surgery and approximately 4 days thereafter using multi-slice (at least 64 slices) scanners in the helical scanning mode. Iodinated contrast media was administered intravenously with a power injector automatically timed to the arterial phase. The axial images were reconstructed with a slice thickness of 1 mm. Three vascular surgeons performed a review of the MDCT images using the iNtuition 3D workstation (TeraRecon Inc., San Mateo, CA). The region of interest (ROI) was selected as the upper edge of the L5 vertebral body to the lower edge of the pubic symphysis, which was determined on the sagittal images (Fig. 1).

Arterial volume within the ROI was measured using the following segmentation technique. A seeding point was selected in the enhanced artery, the volume of which was automatically measured using a region-growing method.^[10]^ This measurement was repeated for each segment of the enhanced arteries (which were separated by artefacts, including the embolization coil) from the proximal aorta to the peripheral arteries, as long as the Hounsfield units (HUs) were above the standard for enhanced arteries. Peripheral arteries that were not automatically distinguished from adjacent bones because of a similar HU were excluded.

**Figure 2:**
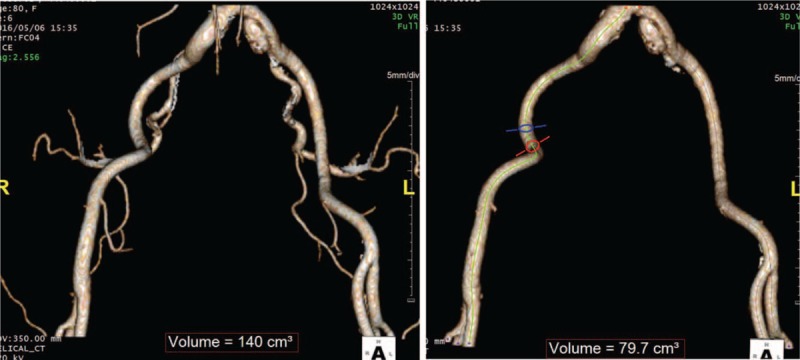
The vascular volume before and after the interventional occlusion of the hypogastric artery.

The sum of the segmented arterial volume was defined as the total arterial volume (TAV). This calculation was performed using axial, sagittal, coronal, and 3-dimensional images. The aortoiliac arterial volume (AIAV) was defined as the sum of the volume of the aorta and the common iliac, external iliac, and femoral arteries and was measured using the automatically extracted centreline of each artery within the ROI (Fig. 2). The difference between the TAV and AIAV was defined as the pelvic arterial volume (PAV), and the ratio of the preoperative PAV to the postoperative PAV (PAV ratio) was assessed. The intraclass correlation coefficient (ICC) was calculated to assess the reproducibility of the PAV measurement between observers.

### 2.5 Statistical analysis

Statistical analysis was performed using JMP 9.0 software (SAS Institute Inc., Cary, NC). Continuous data are expressed as mean ± standard deviation or standard error. Group differences were evaluated using Student *t* test for continuous variables and the chi-squared test for categorical variables with a sample size of more than 4 patients in each category. A *P* value <.05 was considered significant.

## 3 Results

In Study 1, postoperative BC was observed in 16 of 41 patients. Patient data are shown in Table 1A. Hypertension was significantly more prevalent in the non-BC group than in the BC group. Significantly more coils were used for the IOHA in the BC group than in the non-BC group (8.6 ± 1.0 vs 5.6 ± 0.83, *P* = .013) (Table 1B). Furthermore, the percentage of coil insertion into the internal iliac branches was significantly higher in the BC group than in the non-BC group (11 (69%) vs 7 (28%), *P* = .01).

**Table 1:**
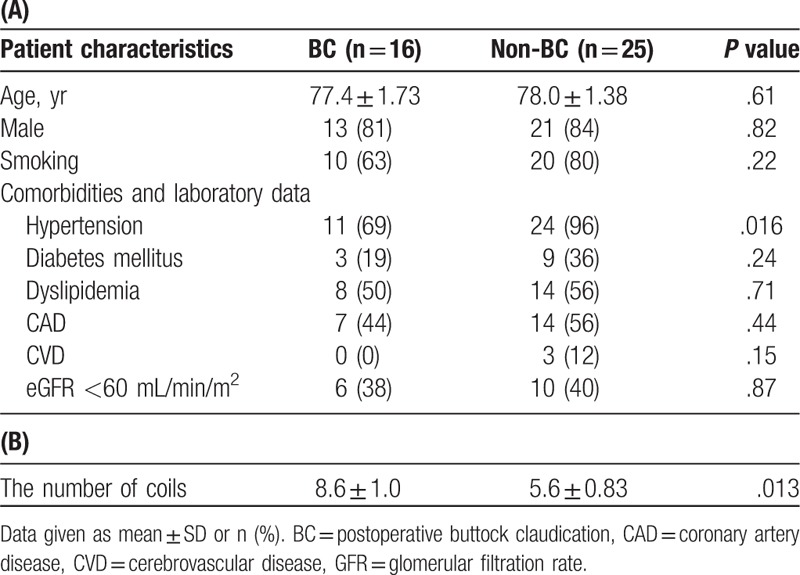
Patient data for Study 1, including the number of embolization coils used for the interventional occlusion of the hypogastric artery.

In Study 2, the average time between the EVAR and postoperative MDCT scan was 4.4 ± 1.8 days. Postoperative BC was observed in 24 of 55 patients. Patient data are shown in Table 2A. The non-BC group had a significantly higher incidence of coronary arterial disease and cerebrovascular disease than that in the BC group. Hypertension was more prevalent in the non-BC group than in BC group; however, the difference was not significant. The proximal IOHA significantly reduced the incidence of postoperative BC compared to that for the distal IOHA (Table 2B). The postoperative MDCT images of 14 patients (45%, 14/31) treated with the proximal IOHA, as well as those of all patients treated with the distal IOHA, showed occlusion of the hypogastric arterial bifurcation into the posterior and anterior branches. Among the patients treated with the proximal IOHA, the rate of this finding in the BC group (42%, 5/12) was comparable with that in the non-BC group (32%, 8/25; *P* = .56). Unilateral IOHA was performed in 47 patients, and bilateral IOHA was performed in 8 patients.

**Table 2:**
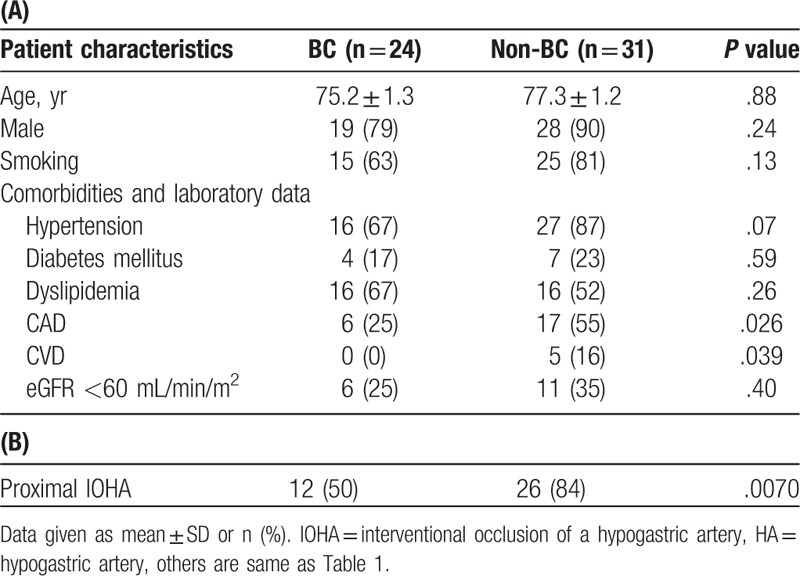
Patient data for Study 2, including the volumetric data.

The inter-observer ICC for the PAV ratio was 0.65, reflecting substantial agreement.^[11]^ Thus, the average PAV ratio across the 3 examiners was evaluated for group differences. The PAV ratio was significantly higher in the BC group than in the non-BC group among the total sample (0.93 ± 0.05 vs 0.62 ± 0.04, *P* <.0001) and among the patients who underwent unilateral IOHA (0.95 ± 0.05 vs 0.64 ± 0.05, *P* <.0001) (Table 3). The PAV ratio among patients who underwent bilateral IOHA did not significantly differ between the BC and non-BC groups.

**Table 3:**
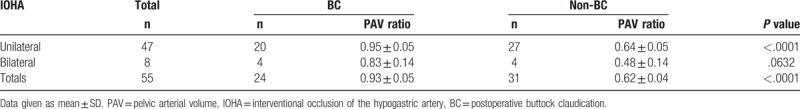
Pelvic arterial volume ratios.

Based on a 2-sample t-test, 41 patients in Study 1 and 55 patients in Study 2 have a statistical power of 80% to detect an effect size of approximately 0.8 (= difference/standard deviation) under an alpha of 0.05. An effect size of 0.8 is considered large^[12]^; thus, this study was fully powered as an exploratory study.

## 4 Discussion

In this study, an interesting and unexpected result was observed. We hypothesised that collateral vessel development would be more pronounced in the non-BC group relative to that in the BC group. However, the present results are completely opposite to our hypothesis. Hypothetically, non-BC patients may have a potential reservation capacity against acute pelvic ischaemia, whereas patients with BC might have less tolerance against acute ischaemia, which triggers collateral vessel development via molecular or neural signals.

If this is true, the strategy for a bilateral IOHA should be changed. For patients with greater capacity, we need not be as concerned about the risk of pelvic ischaemia due to the IOHA, even when performed bilaterally. In contrast, patients with lesser capacity should be recognised as at risk for pelvic ischaemia and should be treated with as few coils as possible, with recommendations for rehabilitation after the operation.

In our department, the interventional procedure for EVAR requiring bilateral IOHA involves 2 steps: we initially perform the ipsilateral IOHA with the EVAR and then perform the contralateral IOHA at least 2 weeks later. If poor collateral development is observed after the initial IOHA, we might predict a good outcome without the adverse events related to pelvic ischaemia.

The proximal IOHA is a critical method of preserving the collateral flow between HA branches and reduces BC as previously reported.^[1,13,14]^ However, the postoperative occlusion of the hypogastric arterial bifurcation after the proximal IOHA is not associated with BC, suggesting that some patients with sufficient pelvic reserve capacity might not need postoperative collateralization contributed by the communication of HA branches. From an economical point of view, the cost of endovascular procedures has increased in recent decades. As there is no clear standard on the number of the coils to use, this depends on the operators’ decision. We made it a rule to use 5 coils or less for a basic IOHA and then added coils only until we confirmed the delayed flow of the contrast media into the HA. This strategy is acceptable, given the lower cost of fewer coils and consequently the lower risk of BC. Although the exclusion effect of embolization should be followed afterwards, the incidence of IOHA-related type 2 endoleaks should be low.^[4,15]^ Some authors have even reported good outcomes using only the exclusion of the stent graft leg without the embolization.^[1,7]^

To our knowledge, this study is the first to evaluate large regions of the body, such as the pelvic cavity, using volumetric studies. The measuring method was semi-automatic and was arbitrarily determined. However, the ICC value was reasonable, and inter-observer biases may be minimised. In addition to the TeraRecon software used in this study, numerous other potentially useful software programs exist. Thus, this method may become more readily replicated in the future.

This study has some limitations. First, the study has a retrospective design and involves a single institute. Thus, generalisation to other patient populations may not be possible. Second, it was difficult to evaluate the degree of BC, as the timing of the interview relative to the latest CT varied among patients. In addition to an interview regarding BC, the use of near-infrared spectroscopy (NIRS) on the hip might be needed to evaluate the minute perfusion of the gluteal muscle.^[16]^ Prospective further studies to assess buttock tissue flow using this modality are necessary. Third, the volumetric method was performed only semi-automatically.

In conclusion, more coils were used for the IOHA in the BC group than in the non-BC group. In addition, the volumetric analysis revealed that less collateral vessel development occurred in the non-BC group than in the BC group. These results have implications for surgical decisions that may reduce the incidence of BC and improve the post-EVAR quality of life.

## Author contributions

**Conceptualization:** Katsuyuki Hoshina.

**Data curation:** Jun Nitta, Toshihiko Isaji; Katsuyuki Hoshina.

**Writing – original draft:** Jun Nitta.

**Writing – review & editing:** Katsuyuki Hoshina.
